# Maintenance of color memoranda in activity-quiescent working memory states: Evidence from impulse perturbation

**DOI:** 10.1016/j.isci.2024.109565

**Published:** 2024-03-26

**Authors:** Güven Kandemir, Sophia A. Wilhelm, Nikolai Axmacher, Elkan G. Akyürek

**Affiliations:** 1 Department of Experimental Psychology, University of Groningen, Groningen 9712 TS, the Netherlands; 2 Department of Experimental and Applied Psychology, Vrije Universiteit Amsterdam, Amsterdam 1081 BT, the Netherlands; 3 Department of Neuropsychology, Faculty of Psychology, Ruhr University Bochum, 44780 Bochum, Germany

**Keywords:** Sensory neuroscience, Cognitive neuroscience

## Abstract

In the present study, we used an impulse perturbation method to probe working memory maintenance of colors in neurally active and activity-quiescent states, focusing on a set of pre-registered analyses. We analyzed the electroencephalograph (EEG) data of 30 participants who completed a delayed match-to-sample working memory task, in which one of the two items that were presented was retro-cued as task relevant. The analyses revealed that both cued and uncued colors were decodable from impulse-evoked activity, the latter in contrast to previous reports of working memory for orientation gratings. Decoding of colors from oscillations in the alpha band showed that cued items could be decoded therein whereas uncued items could not. Overall, the outcomes suggest that subtle differences exist between the representation of colors, and that of stimuli with spatial properties, but the present results also demonstrate that regardless of their specific neural state, both are accessible through visual impulse perturbation

## Highlights

• Impulse perturbation reveals task-relevant and task-irrelevant colors in working memory
• Impulse perturbation impacts alpha band activity, revealing currently active memories
• Alpha power decoding reflects selection and maintenance of task-relevant items only
• Color space in memory is mainly circular but discrete representations also contribute

## Graphical abstract

**Figure:**
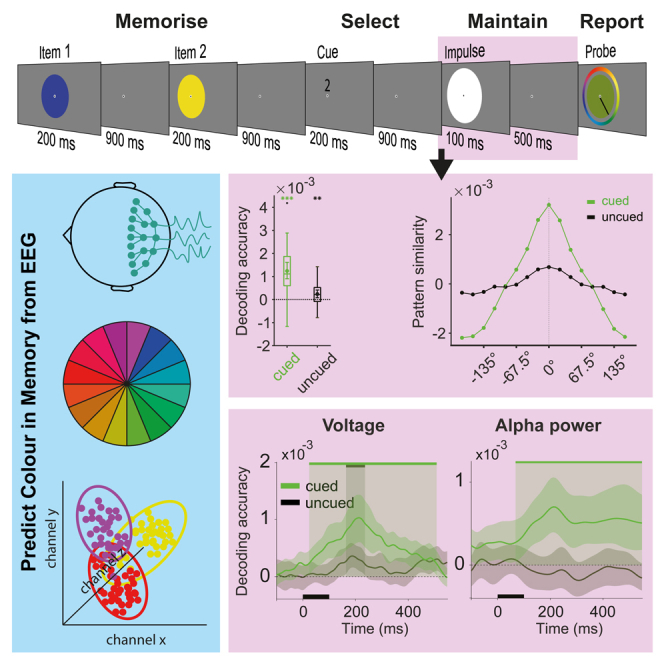

## Introduction

Working memory is a system of components that enables the maintenance of information in an accessible state in the absence of sensory stimulation.^1^ The neural basis of working memory has been of increasing interest in recent years. One particularly striking outcome has been that although working memory maintenance has been traditionally associated with sustained neural activity during the memory delay period,^2^^,^^3^^,^^4^ a number of studies failed to observe such sustained activity.^5^^,^^6^^,^^7^^,^^8^ It was proposed that during this period without an observable neural correlate, memoranda could be retained in activity-silent states,^9^^,^^10^^,^^11^ which may rely on synaptic plasticity facilitated by elevated post-excitatory calcium and neurotransmitter levels.^12^^,^^13^^,^^14^^,^^15^^,^^16^^,^^17^

Wolff and colleagues^11^ have previously shown that the presentation of a high-contrast, standardized, but task-irrelevant stimulus during the memory delay may allow decoding of such activity-silent, or at least activity-quiescent, memory items that could not be detected from raw ongoing electroencephalography (EEG). While it is not yet exactly clear how this so-called impulse signal reveals the memory trace at a physiological level, Wolff and colleagues^11^ explained this using the analogy of sonar: the impulse allows measuring a “hidden” state by attributing differences in the response to a stable stimulus to underlying differences in the network.^11^ In our case, the sensory processing of an impulse signal is thought to perturb the initially “hidden” memory network, generating activity that can be measured with EEG, from which the state of this network can be inferred.^9^^,^^11^^,^^18^^,^^19^^,^^20^ Studies using the impulse perturbation approach have reported successful results for memories of orientations,^11^^,^^18^^,^^19^^,^^20^ numerosity,^21^ and auditory tone frequencies and sequences.^19^^,^^22^^,^^23^

Although impulse perturbation has thus been used with different stimuli, it is yet uncertain whether the representational patterns observed to date will hold universally for different kinds of content that are maintained in working memory. In particular, one crucial similarity between previously tested memoranda is that they allow a transformation of the task-relevant information into spatial coordinates (which may also aid memory). For example, each member of a set of orientations can be represented as different points on the edge of an imaginary circle around a fixed point on a plane surface. In a similar fashion, higher or lower numerosity and tone frequency can be easily converted to different elevation levels laid out on a similar surface (e.g., a high tone may be visualized high on the vertical axis). This possibility of representing information with spatial position leaves open the question of whether the neural signature of the maintenance of this kind of information also applies to that of non-spatial stimulus attributes.

The reliance on spatial properties in previous studies also brings confounding risks with it. For example, it might involve the deployment of spatial attention, which might affect the alpha band of the EEG in particular.^24^ Bae and Luck^25^ investigated the contribution of spatial attention to the decoding of orientation items in working memory. They found that while alpha band activity only conveyed information about the (attended) location of their stimuli (but see also Barbosa et al.^26^), and while decoding of ongoing EEG during the delay period mainly provided significant information about the item-specific orientation, it also reflected its location. Furthermore, differences that exist between stimuli or conditions in terms of spatial attention come with the risk that (voluntary or involuntary) eye movements may follow suit. Neural data can be confounded by correlating eye movements and gaze fixations, especially under active viewing conditions.^27^^,^^28^^,^^29^ Activity in the brain, particularly in earlier visual regions, may reflect viewpoint-specific, retinotopically organized information that will vary considerably when the eyes move around. Such activity cannot be easily discerned from other aspects of sensory and cognitive processing, including memory maintenance.^30^^,^^31^

Additionally, a number of studies suggest that spatial properties may be treated differently by working memory.^32^^,^^33^^,^^34^ For example, evidence suggests that task-irrelevant features, such as color or orientation, may not be encoded in memory,^35^^,^^36^ even when the task relevant and irrelevant features spatially overlap. However, the location of a memory item, even when this feature is irrelevant for the task, can still be traced from the EEG data.^37^ Likewise, the spatial position of all memoranda was reflected in persistent activity in alpha band, even when only one of the items was prioritized retrospectively to drive the response.^37^^,^^38^ Thus, the privileged position of spatial information in working memory might also account for impulse-driven decoding.

Considering the theoretical limitations and potential confounding issues with regard to the exclusive reliance on spatial properties in previous impulse-based experiments on working memory maintenance, we set out in the current study to overcome these by using intrinsically non-spatial stimuli, namely colors. Colors can be decoded successfully from fMRI,^39^^,^^40^ MEG (magnetoencephalography),^41^^,^^42^^,^^43^ and EEG data.^35^^,^^43^^,^^44^ Thus, colors seem a suitable non-spatial substitute to extend earlier orientation-based pinging studies.^11^^,^^18^^,^^19^^,^^20^ Apart from thus changing the memory items, we also presented them serially, at the same location, rather than lateralized, as was originally done, and rotated the color wheel that served as the response probe on each trial, thereby removing all spatial aspects from the original task.

We collected EEG data in each trial of our experiment, as the participants viewed two colored discs (200 ms each), a numerical retro-cue (200 ms), a white disc (100 ms) that served as the impulse, each with 900 ms delay in-between, and finally a response screen, after another 500 ms delay. We then conducted a set of pre-registered analyses, based on those reported in the original paper by Wolff and colleagues,^11^ and added decoding analyses of alpha band activity. Our results show that trial-specific color information could be successfully predicted from the activity evoked by the visual impulse, similar to orientation gratings in earlier studies.^11^^,^^18^^,^^19^^,^^20^ In addition to the task-relevant cued color, dynamic impulse-driven activity also revealed the uncued color. Conversely, while alpha power decoding yielded a sustained trace of the cued memory item, this was not the case for the uncued color. These findings extend previous work on orientation decoding, and suggest that different features might elicit (slightly) different maintenance mechanisms. The present outcomes also highlight that impulse-driven decoding can reveal memoranda in distinct memory states, independent of the allocation of spatial attention.

## Results

### Behavioral results

**Figure 1:**
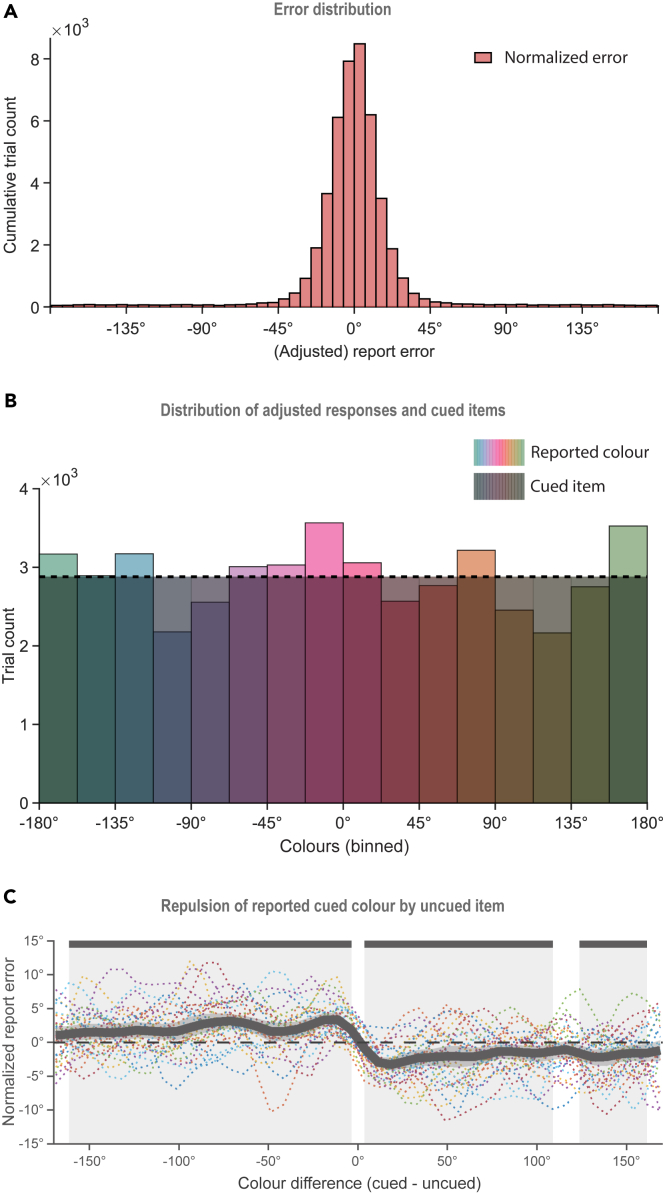
Behavioral plots (A) Histogram of report errors relative to the task-relevant memory item. (B) Distribution of the cued color bins (shaded, dashed line) and the reported color bins, as a function of the angular values of the color space. (C) Normalized error as a function of the difference between the cued and the uncued item. The moving mean of the report error is calculated for 22.5° wide bins in steps of 7.5°. The shaded area and the solid bars at the top mark the color differences for which the adjusted error differed from 0°, according to the cluster-corrected permutation test (p < 0.05).

The overall behavioral performance in the experiment was good; the mean error was 18.3° with a standard deviation of 32.5° (Figure 1A), relative to the center of the bin to which the cued item belonged (adjusted error). Figure 1B presents the behavioral reports of the cued item, with the uniform color bins overlaid. This plot reveals that despite the uniform distribution of the colors presented during the experiment, the reported colors seemed clustered around the primary hues, supporting earlier evidence that memory performance varies for different colors.^45^

The influence of the uncued memory item on the report of the cued item was also investigated. Since only the cued item was reported in each trial, the presence of the uncued item was assessed as the effect of the similarity between the cued and the uncued color on the degree of error. To take into consideration possible individual differences in color perception,^46^ errors were first median-normalized within each color bin. The normalized error values were then binned again as a function of the difference between the task-relevant cued item and the task-irrelevant uncued item (binwidth = 22.5°, moving window in steps of 7.5°), and the mean error was calculated within each bin. A permutation test was applied with cluster correction to assess the deviation of the mean from zero at each unit of angular difference between the items. The report error was significantly different from zero for three difference ranges (Figure 1C, M_Adjusted error_ ≠ 0, for cued – uncued difference, ranging from −161° to −3.75°, p < 0.001, from 3.75° to 116°, p < 0.001, and from 124° to 161°, p = 0.024). The cued item report errors deviated away from the uncued item, reflecting a repulsion away from the task-irrelevant color.

### Decoding the time window of interest

**Figure 2:**
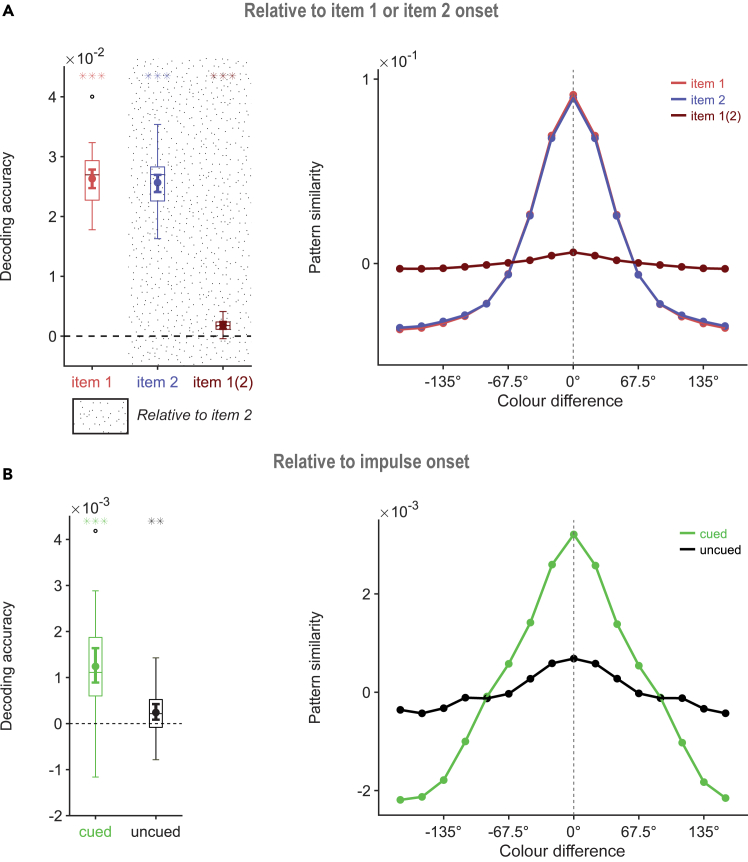
Decoding accuracy and pattern similarity Boxplots and tuning curves showing decoding accuracy and pattern similarity (in arbitrary units) for memory items within the 100–400 ms time window of interest relative to the onset of item 1 and 2 presentation (A), as well as impulse onset (B). The mean decoding accuracy is marked by the dot at the center, with the bar represent 95% CI. The boxes border the 25^th^ and 75^th^ percentiles, with the whiskers around the box stretching to 1.5 interquartile range. Asterisks indicate beta values that were significantly above zero (∗, p < 0.05; ∗∗, p < 0.01; ∗∗∗, p < 0.001). (See Figure S1 for the topographical distribution of the most contributing electrodes).

Both the trial-specific color of item 1 (Figure 2A, left, red, p < 0.001, one-tailed) and of item 2 (Figure 2A, left, blue, p < 0.001, one-tailed), could be successfully decoded after presentation. Interestingly, item 1 was also decodable within the critical period following the presentation of item 2 (Figure 2A, left, item 1 (2), red, p < 0.001, one-tailed). Decoding of item 1 after the onset of item 2 was nevertheless significantly lower than stimulus-driven decoding (difference _item 1 – item1 (2)_, p < 0.001, one-tailed). The associated tuning curves (Figure 2A, right) reflected a parametric-looking relationship between colors, in line with earlier reports.^44^

After impulse presentation, not only the task-relevant, cued color was decodable (Figure 2B, left, green, p < 0.001, one-tailed) but also the task-irrelevant uncued item (Figure 2B, left, gray, p = 0.008, one-tailed), in contrast to earlier studies that used orientation stimuli (e.g., Wolf et al.^11^). Although both cued and uncued memories could be traced, there was a clear difference in the strength of their representations (difference _cued – uncued_, p < 0.001, one-tailed). Both the cued and uncued item showed parametric-looking pattern similarity, as expected (Figure 2B, right).

### Time-course decoding

In addition to classic time-course decoding of colors, we additionally applied this analysis to alpha power. Motivating this addition was the recent suggestion that ongoing activity in the alpha band could reflect memory content in a sustained fashion, which questions the need to use the impulse perturbation method.^26^ The analyses were focused on the cue and impulse epochs, where the selection between task-relevant (cued) and task-irrelevant (uncued) items had been made. Furthermore, we were able to confirm that eye movements, attentionally driven or otherwise, did not affect decoding of the memory items by applying the same analysis to the electrooculography (EOG) data (supplemental information).

**Figure 3:**
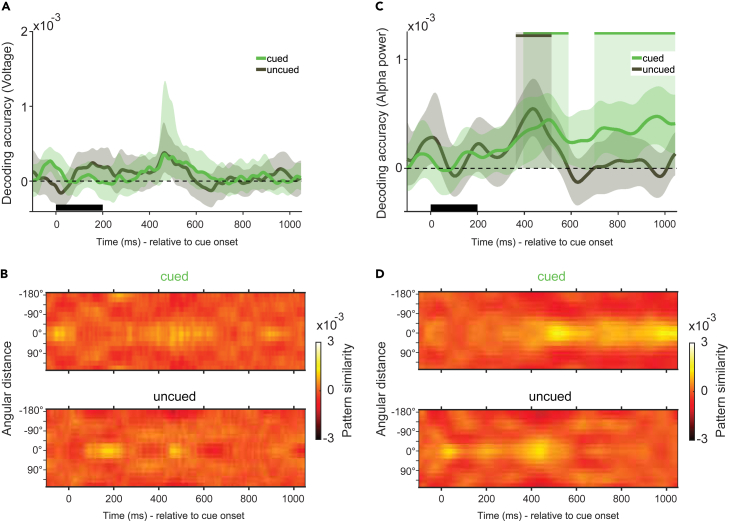
Time-course decoding in cue epoch The mean decoding accuracy of the cued (green) and the uncued (black) item relative to the onset of the cue from the raw voltages (A and B), and from alpha power (C and D). (A and C) The black rectangular bar marks the presentation of the cue. Solid lines show the mean decoding accuracy (A.U.) over all trials and participants as a function of time. The shaded area around the mean marks the 95% CI. Solid bars at the top and the shaded zones indicate statistically significant decoding periods (p < 0.05, one-sided). (B and D) Pattern similarity matrices for cued and uncued items show reverse-signed, mean-centered Mahalanobis distances between the target item and all other possible memory items, averaged over trials as a function of time. (See Figure S2A for an analysis of the same epoch from the eye electrodes).

The memory items could not be decoded from the voltage data following the presentation of the cue (Figures 3A and 3B). Conversely, both the cued and the uncued items were successfully decoded from alpha power in an earlier phase after the presentation of the cue (Figure 3C, green, 396 ms–588 ms, p_*cued*_ = 0.015, two-tailed, corrected; Figure 3C, black, 364 ms–516 ms, p_*uncued*_ = 0.014, two-tailed, corrected). Subsequently, in a later phase, only the task-relevant, cued color was decodable in the alpha band without interruption for the remainder of the epoch until the onset of the impulse signal (Figure 3C, green, 700 ms–1050 ms, p_*cued*_ < 0.001, two-tailed, corrected). The difference in decoding accuracy between cued and uncued items was also statistically significant for some time within this period, from 892 ms to 1,028 ms relative to impulse onset (p_*cued - uncued*_ = 0.037, one-tailed, corrected). Across these phases, the pattern similarity appeared to be parametrical in nature for both items (Figure 3D).

**Figure 4:**
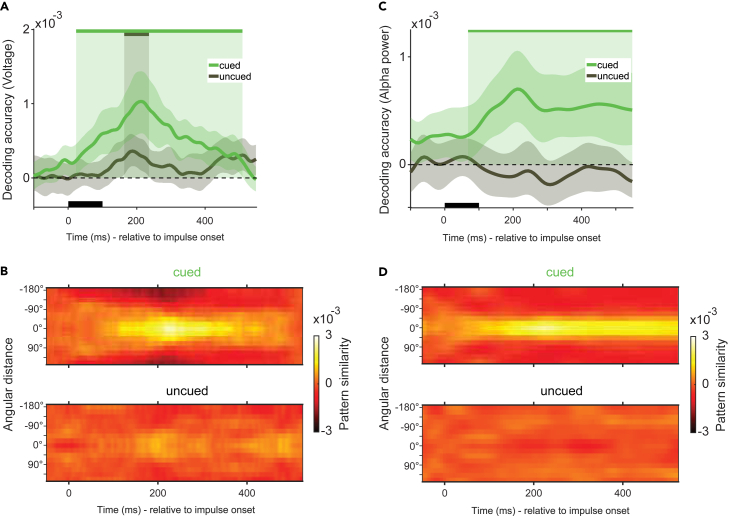
Time-course decoding in impulse epoch The mean decoding accuracy of the cued (green) and the uncued (black) item relative to impulse onset from the raw voltages (A and B), and from alpha power (C and D). (A and C) The black rectangular bar marks the presentation of the impulse. Solid lines show the mean decoding accuracy (A.U.) over all trials and participants as a function of time. The shaded area around the mean marks the 95% CI. Solid bars at the top and the shaded zones indicate statistically significant decoding periods (p < 0.05, one-sided). (B and D) Pattern similarity matrices for cued and uncued items show reverse-signed, mean-centered Mahalanobis distances between the target item and all other possible memory items, averaged over trials as a function of time. (See Figure S2B for an analysis of the same epoch from the eye electrodes).

At impulse presentation, voltage decoding revealed both the cued (Figure 4A, green, 28 ms–514 ms, p_*cued*_ < 0.001, two-tailed, corrected), and the uncued item (Figure 4A, black, 164 ms–236 ms, p_*uncued*_ = 0.036, two-tailed, corrected). The difference between the states of these two items was reflected by a significant difference in decoding accuracy (118 ms–356 ms, p_*cued - uncued*_ = 0.005, one-tailed, corrected). Pattern similarity reflected this difference also, but was qualitatively similar for both items (Figure 4B). These results confirmed the outcomes of the analysis of the time window of interest reported above. The cued item was also decodable from alpha power (Figure 4C, green, 68 ms–550 ms, p < 0.001, two-tailed, corrected), but the uncued item was not (difference; 92 ms–548 ms, p_*cued - uncued*_ < 0.001, one-tailed, corrected). The pattern similarity matrix of the cued item showed high similarity near the tested values, with a steep drop-off further away (Figure 4D). These results suggest that while alpha power may reflect the sustained maintenance of, or the attention allocated to, the task-relevant memory item, the task-irrelevant item only emerged from the activity evoked by the impulse. Given that the uncued item also biased the eventual behavioral response, the present findings provide evidence that the impulse signal can reveal memory items retained in different activity states.^9^^,^^47^

### RSA of color representations

**Figure 5:**
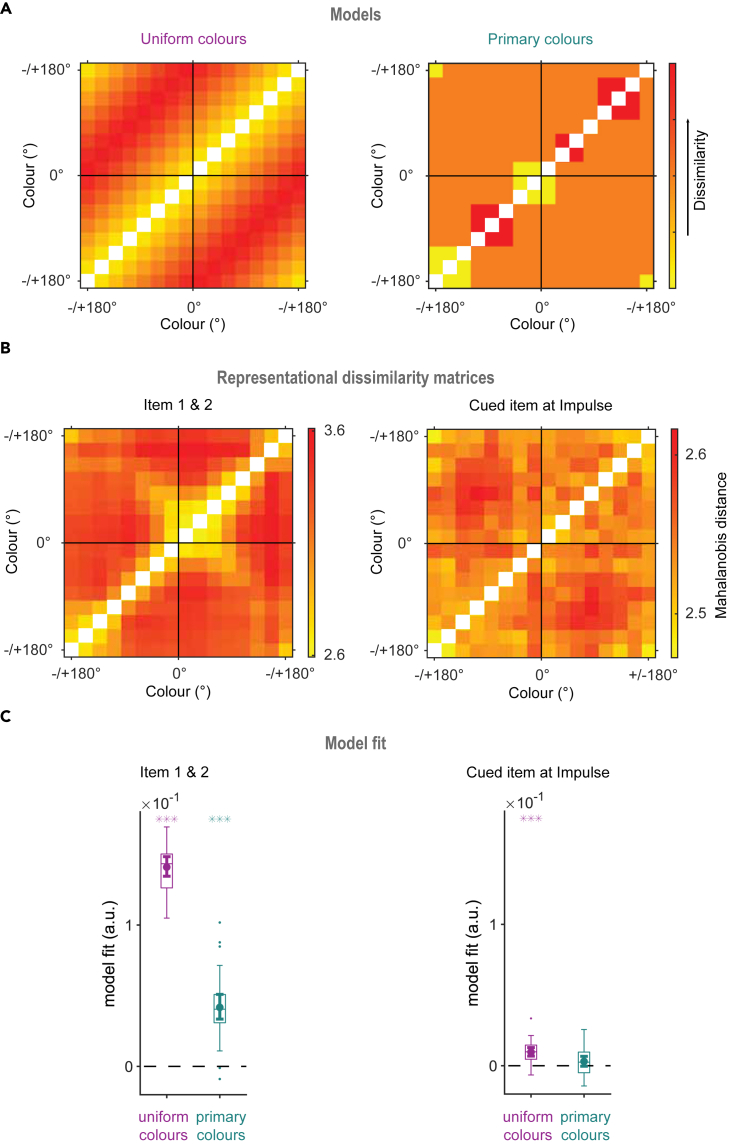
Representational similarity analysis Analysis results for the average of item 1 and 2 (left) and the cued item at impulse (right). (A) Model for a uniform, continuous color space for 16 color bins (left), and model for a discrete color space based on primary colors extracted from behavioral errors (right). (B) The representational dissimilarity matrices (RDM) for item presentation (left) and impulse (right) epochs. (C) Boxplots showing the mean fit (beta values) following the linear regression of individual RDMs on the models for each participant. The dot in the center marks the mean standardized slope (beta), and the whiskers on both ends of the mean indicate the 95% CI. The boxes border the 25^th^ and 75^th^ percentiles, with the whiskers around the box stretching to 1.5 interquartile range. Asterisks indicate beta values that were significantly above zero (∗, p < 0.05; ∗∗, p < 0.01; ∗∗∗, p < 0.001).

Finally, we investigated the color-space with a parametric method, representational similarity analyses.^19^^,^^48^ Two models were tested to explain pairwise differences between color bins. The first model was based on a uniform color space that reflected the angular differences between 16 color bins on the color wheel (Figure 5A, left). This model tested whether colors adhered to a circular, parametric space. The second model was based on the behavioral reports (Figure 5A, right), in which reported colors were found to cluster around three primary colors, despite the uniform distribution of memory items (Figure 1B). In the model, these three colors were thus used to explain the variation in the data. The average representational dissimilarity matrices (RDMs) presented in Figure 5B reflect the pairwise differences between the 16 color bins within the time window of 100–400 ms, relative to the onset of the memory items (left), and relative to impulse onset (right).

Both models fit the data during stimulus encoding (Figure 5C, left, p_*Uniform colors*_ < 0.001, one-tailed; p_*Primary colors*_ < 0.001, one-tailed). Thus, the results provided evidence for a parametric relationship between color representations during sensory encoding, as well as a degree of primary-color categorization. The uniform color space model also fit the neural response associated with the cued item that was evoked by the impulse (Figure 5C, right, p_*Uniform colors*_ < 0.001, one-tailed), in line with earlier reports.^44^ Conversely, although there was a trend, the primary colors model failed to reach statistical significance (Figure 5C, right, p_*Primary colors*_ = 0.054, one-tailed).

## Discussion

We aimed to decode colors maintained in working memory by means of visual impulse perturbation. In a pre-registered experiment, we tested the maintenance of a retro-actively cued target item, and of the item that was not cued, in a delayed match-to-sample task. To further avoid possible spatial confounds that might have affected previous impulse perturbation experiments, we presented our two memory items serially in the center of the screen, and randomized the appearance of the color circle shown at the probe on each trial. We were able to decode stimulus identity from the recorded EEG signal during perceptual encoding, replicating earlier studies.^35^^,^^39^^,^^40^^,^^41^^,^^42^^,^^43^^,^^44^ Importantly, we could also decode stimulus identity post-impulse. This result extended earlier studies that decoded the bottom-up activity induced by the sensory processing of a visual impulse signal to reveal representations of orientation gratings in working memory that were otherwise not traceable from raw EEG (e.g., Wolff et al.^11^^,^^18^^,^^20^). The present result was the first demonstration of impulse-driven decoding of non-spatial features, specifically color memories, thereby generalizing the findings across feature dimensions.

In contrast to previous studies,^5^^,^^6^^,^^11^^,^^19^^,^^20^^,^^49^ the impulse effect was not restricted to the task-relevant cued item. This suggests that after its original encoding, and after the cue designating it as task-irrelevant, the uncued item was also maintained in memory, albeit to a lesser degree. One trivial reason for this might be that participants simply selected the wrong item on some trials, but there is reason to doubt this explanation. First, in alpha power decoding (discussed in the following text), the signal associated with the uncued item behaved completely different from the cued item, both during maintenance and following the impulse. Second, behavioral response errors on the cued item were biased by the uncued item (cf. previous studies^50^^,^^51^^,^^52^), but they were biased away from it, rather than toward it—the latter would be expected when the uncued item was erroneously reported. The presence of this bias also suggests that the uncued item did not elicit an impulse response simply because it was previously presented and perceived (i.e., without being committed to memory).

The emergence of the uncued item after impulse onset casts doubt on the idea that information in working memory that is no longer needed is actively purged. Memory might alternatively “let go of” task-irrelevant items, for instance by no longer periodically refreshing them, such that their representations fade relatively quickly, but might nevertheless still linger for some time. This idea is compatible also with a recent model of working memory based on calcium-mediated short-term synaptic plasticity.^53^ The absence of task-irrelevant items in neural measures obtained in previous studies might be a consequence of the inherent weakness of their representations, which makes them harder to detect than task-relevant items in the first place. The high number of trials in the current study may have helped to overcome this difficulty.

Alternatively, colors may be represented differently in memory. It is possible that they are more strongly represented in long-term memory, and/or with more distributed connections, as they are well-known entities, in comparison to orientations that are derived from relatively novel Gabor stimuli used in other studies (e.g., Wolff et al.^11^^,^^19^). This difference may also cause the uncued item to be reflected in the impulse response in this study, but this remains speculative. From the present data, it cannot be excluded either that the presence of the uncued item during memory maintenance might be related specifically to the metathetic nature of color. This awaits further experimentation.

The decoding analyses showed that alpha band decoding and dynamic voltage decoding clearly reflected different states of the memoranda. After cue onset, item selection following the processing of the cue was only observable in alpha power. Both the task-relevant and irrelevant colors could initially be decoded, followed by a sustained signal for the cued item only. With regard to the cued item, the presentation of the cue would be hypothesized to require an active process of prioritizing one item over the other or potentially even removing the uncued item from memory storage (but see the argument aforementioned). Even theories of activity-silent working memory have so far proposed connectivity-based, synaptic storage only as a mechanism of maintenance, and updating of synaptic weights would still require neuronal firing.^9^^,^^10^^,^^14^^,^^53^^,^^54^^,^^55^ Based on the analyses of our current dataset it appears that this attentional selection happens in the alpha band, which subsequently then also holds the prioritized item in a sustained manner for the rest of the trial. With regard to the uncued item, the absence of a sustained signal both in the raw EEG and in the alpha band suggests that it was truly activity quiescent, if not altogether silent, as previous work has identified the alpha band as the frequency band that is most likely to carry sustained maintenance signals.^26^^,^^56^

Once the impulse was presented, the voltage decoding revealed both the cued and uncued item, contrary to the alpha band decoding. Additionally, the reactivation of the signal in the voltage decoding was time limited and returned to zero well before the presentation of the probe. This provides further evidence that the alpha band and voltage decoding track functionally different states of working memory. We speculate that the alpha band may serve to keep memoranda in an elevated state, ready for direct access when necessary.^57^^,^^58^ It seems likely that attention mediates this.^25^ Following the cue, the uncued item was apparently removed from this elevated state, but it was not altogether lost, as dynamic voltage decoding still revealed it from the EEG response to the impulse. Although the functional states of items in memory need not necessarily map directly onto corresponding neural states,^47^^,^^59^ the task-irrelevant uncued item was clearly in a different, more silent, neural state than the task-relevant cued item in our data. The fact that we can trace these differences and differentiate them provides further support for the utility of the impulse-driven decoding technique.

Finally, we found that the neural representations of colors were parametrically arranged during encoding.^39^^,^^44^ The signal evoked by the impulse also showed a parametric arrangement of colors during the delay period. Nevertheless, behavioral responses reflected a bias in reports, as the errors were grouped around three primary colors. This observation was in line with earlier reports that not all colors are equally memorable.^45^ A discrete color model derived from the behavioral output was indeed also supported during the encoding of the memory items (i.e., directly after their onset), suggesting that participants formed a categorical representation for colors even during perception or shortly thereafter. Crucially, the evidence for this discrete model was no longer reliable during memory maintenance (i.e., after the impulse), while the continuous model was still well supported. It may appear paradoxical that the categorical representation observed during encoding, and in the eventual response, was not clearly represented during memory maintenance. One possible explanation for this could be the differential impact of the activity induced by the sensory processing of the impulse signal on the different networks that might retain continuous color representations and discrete categorical representations. One might suppose that more discrete color groupings could be represented by a semantic network,^42^ whereas more low-level hue differences could be retained in earlier visual areas.^39^ The latter might be more accessible to impulse perturbation.

### Conclusion

The results of our study highlighted both commonalities and potential differences between spatial and non-spatial (color) items maintained in working memory. First, we observed that the visual impulse response contained information not only about the task-relevant, cued item but also the task-irrelevant, uncued item, contrary to previous studies of orientation gratings. This finding casts doubt on the idea that uncued items are actively purged from memory. Second, we found evidence for a sustained signal corresponding to the cued, but not the uncued, item during the delay period. This suggests that the alpha band may trace the attended item that is in the focus of attention. Furthering the debate on the relationship between the functional role of an item and its activity state,^26^^,^^47^^,^^55^ the current results provided evidence for the utility of the impulse technique by revealing silent memories that were not only hidden in ongoing EEG but also untraceable by alpha power.

### Limitations of the study

The experiment was conducted in a well-lit room, using an old uncalibrated cathode ray tube (CRT) monitor. Room light and monitor settings can influence perceived colors and therefore impact decoding accuracy as well. Although these factors would not influence the reported differences in alpha band power and voltage decoding, they could reduce generalizability. Furthermore, the sample of participants mostly consisted of young adult European females studying at the university, which could impact color labeling and strategies used to remember different colors. This factor could also hamper the generalizability of our results in samples from other cultures and genders.

## STAR★Methods

### Key resources table

**Table:** 

REAGENT or RESOURCE	SOURCE	IDENTIFIER
**Deposited data**		

Filtered and epoched EEG data and behavioural response data	This paper	https://osf.io/bxmt8

**Software and algorithms**		

Experiment script	This paper	https://osf.io/bxmt8
Colour wheel	Mem toolbox, Suchow et al.^60^	https://visionlab.github.io/MemToolbox/
Analysis and plotting scripts	This paper	https://osf.io/bxmt8

**Other**		

Preregistration	This paper	https://osf.io/uvxe7

### Resource availability

#### Lead contact

Further information and requests for resources and reagents should be directed to and will be fulfilled by the lead contact, Güven Kandemir (g.kandemir@vu.nl). Any additional information required to reanalyse the data reported in this paper is available from the lead contact upon request.

#### Materials availability

The experiment script used for data collection is publicly available at https://osf.io/bxmt8. Any additional information required to reanalyse the data reported in this paper is available from the lead contact upon request.

#### Data and code availability

• Filtered and epoched EEG data and the associated behavioural data reported in this study have been deposited at the Open Science Framework (OSF) repository. The links to the data and pre-registration documents are listed in the key resources table.
• All original code has been deposited at OSF repository and is publicly available as of the date of publication. The link is listed in the key resources table.
• Any additional information required to reanalyse the data reported in this paper is available from the lead contact upon request.

### Experimental model and study participant details

#### Participants

Thirty volunteers (23 female, M_age_ = 24.2, Range_age_ = 20-36) that were recruited via social media adverts participated in this study in return for monetary rewards. No race, ancestry or ethnicity information was collected for this study. This was justified on the basis that racial differences do not influence colour perception.^61^ Participant selection relied on the successful completion of a pre-screening test, which was a shortened version of the main experiment (288 trials). The preselection cut-off criterion was ≤30° of error in at least 70 % of trials. None of the volunteers were eliminated by the pre-screening. The sample size was based on earlier studies with similar designs (e.g., Wolff et al.^11^). All participants were informed about the experimental procedures as well as the data sharing procedures, and written consent was obtained. The study was conducted in accordance with the Declaration of Helsinki (2008), and it was approved by the Ethical Committee of the Behavioural and Social Sciences Faculty of the University of Groningen (Study ID = PSY-1920-S-0385).

#### Apparatus and stimuli

The experiment took place in a well-lit chamber where participants were seated 60 cm away from a 17” Samsung 797DF CRT monitor. The refresh rate was set to 100 Hz and the resolution was 1024 by 768 pixels. All stimuli were created and presented with the freely available Psychtoolbox 3^62^^,^^60^ extension for Matlab .

The memory items and the probe consisted of coloured disks with a visual angle of 6.69°, which were presented in the centre of the screen. Their colours were randomly drawn from RGB conversions of 48 equiluminant colours equally distanced on the CIELAB colour wheel, which were extracted from the freely available Matlab extension MemToolbox.^63^ A grey background (RGB = 128, 128, 128) was maintained throughout the experiment. A black fixation dot with a white outline (0.25° of visual angle) was displayed in the centre of the screen at all times except during the presentation of the cue. The cue was a number, “1” or “2”, indicating the serial position of the task-relevant (“cued”) item in that trial, presented in Arial font in the centre of the screen (0.5° visual angle). The impulse was a large white disk, displayed in the centre of the screen with a visual angle of 13.38°. The response screen contained the probe in the centre of the screen and a colour wheel surrounding the probe, which had a diameter of 10.05° and a width of 0.55° visual angle. A white line reaching from the centre to the edge of the colour circle indicated the momentary probe colour on the colour wheel. In each trial, the colour wheel was randomly rotated and a random colour was assigned to the probe disk. Responses were collected with an Xbox controller. Following a response, a happy or a sad smiley face was presented at the centre in Arial font, indicating accuracy (i.e., whether the absolute error was less or more than 30°).

### Method details

The overview of a trial is depicted in Figure S1. The experiment consisted of 1536 trials, which were completed in four consecutive sessions that were separated by breaks. Participants determined the duration of the breaks. In each session participants completed 24 blocks, and after each block an average score per block was presented as feedback. Participants could start each block by pressing the SPACE bar, after which trials continued automatically until all the trials in the block were completed. At the beginning of each block, a “Get Ready” warning was presented first, after which the trials commenced.

Each trial started with the presentation of a fixation dot, which was shown for 700 ms on a grey background. This was followed by the serial presentation of two colours for 200 ms duration, each followed by a 900 ms delay. The cue was presented next for 200 ms, and a delay of 900 ms followed it. Next, the impulse signal was presented for 100 ms, and a consecutive delay was on display for 500 ms. The response screen was displayed next and stayed on the screen until a response was submitted. When the response screen was on display, participants could move the left stick on the controller to rotate the black bar presented on the probe, and change the colour of the probe circle. Once the desired colour was selected, the response was submitted by pressing X on the controller. The response was followed by a delay of 150 ms after which feedback was presented for 300 ms. The next trial began automatically after a random jitter with a range of 500 to 800 ms.

### Quantification and statistical analysis

#### EEG Acquisition and pre-processing

The EEG was recorded with Brainvision Recorder software, and a TMSI Refa 8-64/72 amplifier using 62 Ag/AgCl sintered electrodes, which were placed according to the international 10-20 system. The data were recorded in reference to the average of all electrodes at a sampling rate of 1000 Hz. The ground electrode was placed on the sternum, and eye movements were tracked via bipolar electrooculography with vertical electrodes above and below the left eye, and two horizontal electrodes on the ipsilateral sides of both eyes. The resistance at all electrodes was kept below 7 kΩ throughout the experiment.

Filtering and preprocessing were handled via the Matlab extensions Fieldtrip^64^ and EEGLAB.^65^ The data were re-referenced offline to the average of both mastoids. For the multivariate analyses on voltage values, the data were downsampled to 500 Hz, and filtered at 0.1 Hz high-pass and 40 Hz low-pass. Alpha power amplitudes were acquired by bandpass filtering the EEG signal with a 8 Hz high-pass and 12 Hz low-pass, by applying the Matlab function presented below to the data on each channel:

abs(hilbert(eegfilt(data, sample_rate, low_pass, high_pass)));

where low_pass and high_pass corresponded to the 8 and 12 Hz filters applied to our EEG data, and sample_rate corresponded to the 500 Hz sampling rate. The filter output was Hilbert transformed and the absolute of the product was calculated to get the real values of the transformation.

The voltage data and alpha power amplitudes were separately epoched to the onset of the memory items, the cue, and the impulse, covering a range starting from -150 ms relative to their onset until the onset of the next stimulus, thus forming distinct epochs for item 1, item 2, cue and impulse. Semi-automatic artefact rejection was completed by marking trials with high voltage variations and then inspecting all trials visually for channel drifts, muscle and eye artefacts. Drifting channels were interpolated using the spherical head model, whereas epochs with other artefacts and blinks were excluded from the analyses. In total 13.24% of the Item 1 epochs, 10.7% of the Item 2 epochs, 10.76% of the Cue epochs, and 9.99% of the impulse epochs were excluded.

#### Multivariate analyses

Unless stated otherwise, all analyses were pre-registered at https://osf.io/uvxe7/. The decoding analyses were restricted to the 17 posterior channels (P7, P5, P3, P1, Pz, P2, P4, P6, P8, PO7, PO3, POz, PO4, PO8, O1, Oz and O2), replicating earlier studies that investigated visual working memory by means of impulse perturbation.^11^^,^^19^^,^^20^

##### Analysing the time window of interest

The primary analysis aimed to investigate the accuracy of trial-specific colour decoding, which was calculated using the data within a time window of interest. This time window of interest covered 100-400 ms relative to the presentation of a stimulus (e.g., memory item or impulse). This window was based on earlier studies, in which it was applied to capture the bulk of the EEG response to the eliciting stimulus, and in particular the dynamic response to the impulse.^11^^,^^18^^,^^19^^,^^20^ First, the data were baselined by subtracting the average activity within the aforementioned period, which was done separately for each trial and electrode. Next, the data were downsampled by calculating the moving average over a 10 ms window. These downsampled values were then pooled over all 17 posterior electrodes, which yielded a single spatio-temporal pattern for each trial.

The trials were assigned to the closest one of 16 equidistant bins that covered the pre-determined colour-space, and were re-labelled with the centre value of that bin. This was repeated three times to cover the pre-determined colour-space (from 0° to 337.5°, 7.5° to 345°, and 15° to 352°, each in steps of 22.5°), thus providing 16 different colour conditions in each of the three runs. Next, the trials were partitioned into 8 folds with seven folds serving as the training set. Within the training set, the number of trials in each colour condition were equalized by subsampling the data. Subsequently, the data within each colour condition were averaged, forming 16 condition-specific spatio-temporal patterns. These spatio-temporal patterns of colour conditions were then convolved with a half cosine basis set raised to the 15^th^ power to reduce noise and pool information across similar colours.^20^ The similarity between test trials and the averaged training data was quantified in Mahalanobis distances,^66^ yielding 16 distance values for each test trial. The covariance matrix was estimated from the entire training set by using a shrinkage estimator.^67^ The distance values were mean-centred and sign-reversed, so that positive values indicated higher similarity. Finally, the values were convolved with a cosine-similarity function of the colour space (i.e., on the colour wheel). The product was the trial-specific decoding accuracy. The procedure was repeated 100 times with random folds and random sub-sampling, in order to avoid sampling biases. The reported decoding accuracy for each participant was calculated by averaging all the products of all repetitions and all trials to get a single value per participant.

##### Time-course analyses

The time-course of the memory-related dynamic signal was investigated by sliding a 100 ms time-window across the epoch.^20^ In each step, data within the window were baselined by subtracting the mean activity within, and then the residual activity was downsampled to 100 Hz. The data were then pooled over electrode space, yielding the spatio-temporal pattern at that time point. The time-course analysis was highly similar to the analysis of the time window of interest. The trials were first re-labelled in accordance to the colour-space. The trials were divided into 8 folds with stratified sampling so that all conditions had an approximately equal representation in each fold. The training was set formed by 7 folds and the trials in these folds were distributed across 16 bins. The number of trials in each bin was equalized with random subsampling in order to avoid bias, and the data in each bin was averaged to form temporal patterns for each colour bin at each time point. These patterns were smoothed by convolving it with a half cosine basis set raised to the 15^th^ power.^20^ The measure of similarity between the test trials and the 16 averaged colour patterns was calculated at each time point in Mahalanobis distances. A shrinkage estimator^67^ was used to estimate the covariance matrix from the training set. The distance measures at each time point were reverse-signed and mean-centred, and then scaled by cosine convolution of the modelled colour space. The output was the decoding accuracy at each time point. The procedure was performed 3 times, once for each colour-space, and repeated 100 times to avoid sampling bias. The output was averaged over colour spaces, repetitions and trials for each participant.

Post-hoc, we conducted another time-course analysis on (absolute change in) alpha power, which was not included in the preregistration. The analysis was identical to the above, but applied to filtered 8-12 Hz EEG data, which was baselined over -200 to 0 ms relative to cue and impulse onset. The data were also downsampled to 125 Hz to save computational time. Finally, we also conducted the same time-course analysis on the EOG data to ensure the absence of any spatial correlation.

##### Representational similarity analyses

Representational similarity analyses^48^ (RSA) was used to parametrically assess the relationship between colours during memory encoding and during the memory delay following the impulse (using a similar method as Wolff et al.^19^). For the RSA, the EEG data from the same pre-determined 17 posterior electrodes within the time window (100 to 400 ms relative to a stimulus or an impulse signal onset) were prepared as in the decoding analysis of the time window of interest. The trials were re-labelled according to the first colour-space (0° – 337.5° on the colour wheel) and grouped into 16 bins. The number of trials in each bin were equalized and 16 spatio-temporal patterns were generated by averaging trials in each bin. The pairwise differences between all bins were calculated in Mahalanobis distances to form the representational dissimilarity matrix (RDM). The covariance matrix was estimated from the training set with the use of a shrinkage estimator.^67^ The entire procedure was repeated 100 times to account for selection biases and the final RDM was averaged over all repetitions for each participant.

The RDM was tested by the linear regression of two models. The first model covered the circular nature of 16 colour bins centred according to the first colour space. In other words, this model specifically assessed the hue differences in terms of angular space on the colour wheel. The second model was based on the behavioural output, which showed that the responses clustered around three primary colours. The second model thus assessed if the differences in neural space for the 16 colour bins could be explained by the differences between the three frequently reported discrete colours (green-blue, red and yellow). Both models were first converted to z-scores. Next, the RDM of each participant was regressed against each model separately. The diagonal segment for the RDM and models was excluded from analyses. The standardized slopes (beta values) were taken as an indication of the fit. The mean beta values were contrasted against zero by using a group permutation test.

#### Statistical assessment

Trial-wise decoding accuracy was averaged for each participant, yielding a single output (at each time point) per participant. The average decoding accuracy over all participants was contrasted against a null distribution (one-sided). In order to form this null distribution, the sign of the mean decoding accuracy (per participant) was flipped 100,000 times with 50 % probability, and the resultant mean was taken. The proportion of the null distribution larger than the observed mean accuracy was calculated as the *p* value, which was labelled as significant if it was smaller than 5% (*p* < 0.05). For the time-course analysis, an additional cluster correction was applied to control for multiple comparisons, where the cut-off was set to 0.05 (*p* < 0.05). Confidence intervals were built by bootstrapping the mean (n_perm_ = 100,000). When decoding accuracies for two conditions were contrasted, the difference of these two conditions was calculated for each participant, and this group vector was tested against zero by using the same sort of permutation test as explained above. The RSA was similarly assessed by means of a group permutation test (n_perm_ = 100,000), which was applied on the beta values of the participants, reflecting the model fit to the pairwise differences reported in the RDMs.
