# Supplementary material for: Maintenance of color memoranda in activity-quiescent working memory states: Evidence from impulse perturbation
Source: iScience. 2024 Mar 26;27(4):109565. doi: 10.1016/j.isci.2024.109565 (PMC11015458; doi:10.1016/j.isci.2024.109565)
Supplement: Document S1. Figures S1–S3 [file mmc1.pdf]

iScience, Volume *27*

Supplemental information

Maintenance of color memoranda in activity-quiescent working memory states: Evidence from impulse perturbation

Güven Kandemir, Sophia A. Wilhelm, Nikolai Axmacher, and Elkan G. Akyürek

Supplementary Information

A scalp distribution of the memory-related signal was estimated using a Searchlight technique[1, 2]. For this, data within 100-400 ms window relative to stimulus onset, measured from each electrode and its closest two neighbours, were decoded together, using the same approach as the critical time-window analysis. The cosine-convolved output of this analysis corresponds to the decoding accuracy in each trial for the respective electrode. This method was applied 100 times for all 3 colour spaces and the output was averaged over all trials and repetitions, yielding 62 decoding accuracies for the respective electrodes, per participant. A group permutation test (n_{perm} = 100,000) was applied to determine at which electrodes the decoding accuracy was above zero with statistical significance ($p < 0.05$, *one-tailed*).

The results of the searchlight analyses are presented in Figure S3, where lighter colours indicate higher decoding accuracy, and the coloured electrodes (in green) indicate sensors with statistically significant decoding accuracy ($p < 0.05$). Importantly, the highest decoding accuracy was observed for posterior electrodes, including the 17 we used in our analyses both for individual memory items during visual presentation, and for the cued item following the impulse. These observations are similar to earlier reports using orientation stimuli (e.g., Wolff et al.[2]). For the uncued item, the picture was more diffuse, and contributing electrodes were located above left-parietal and central sites on the scalp.

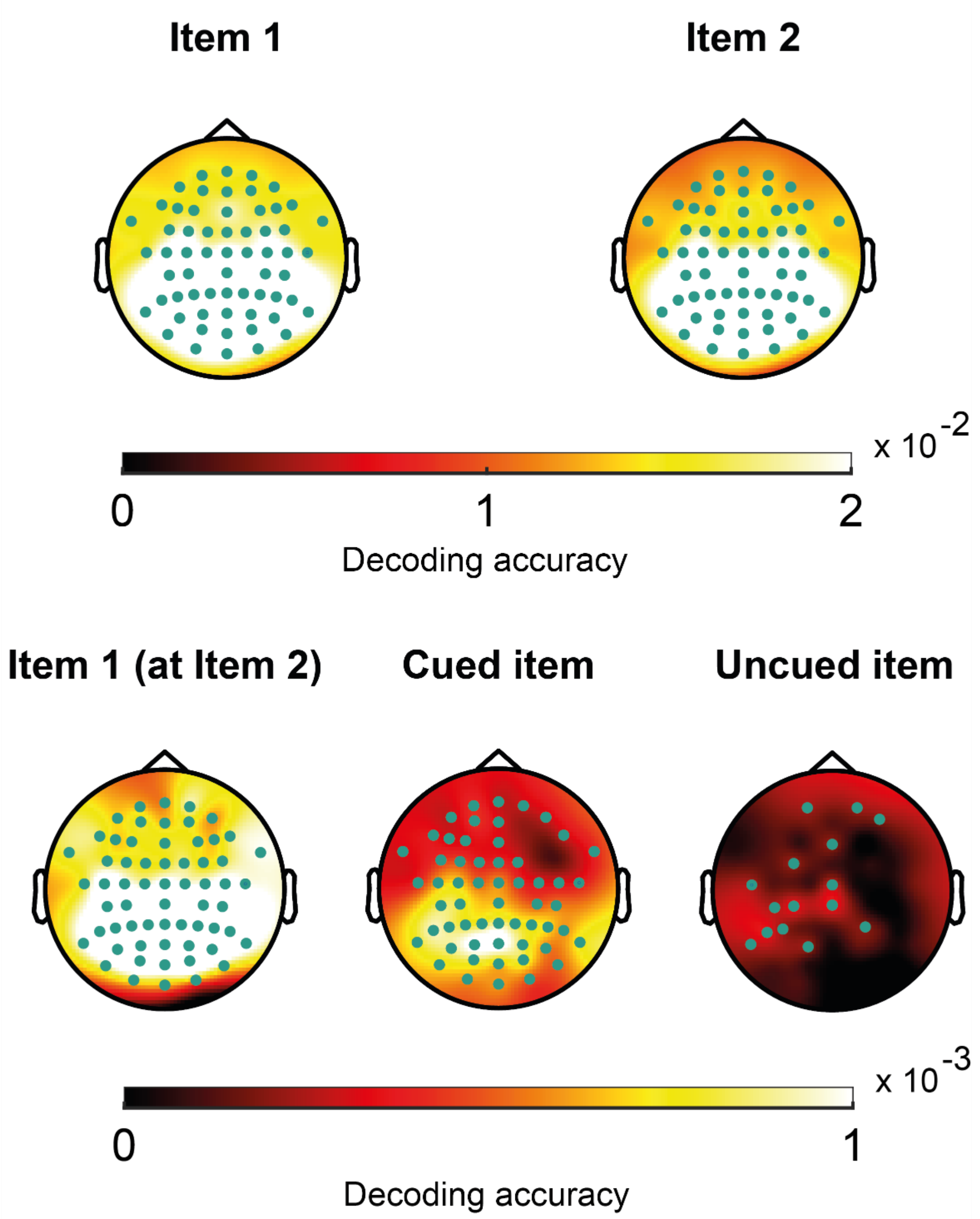

Figure S1. [Topographic plots showing the scalp distribution of the memory signal], Related to Figure 2. Decoding accuracy at each electrode region within 100 – 400 ms window relative to item 1, item 2 and impulse onset. Green dots mark the electrodes with statistically significant decoding ($p < 0.05$, *one-tailed*).

To ensure that the decoding of the memory items was not confounded by (attentionally driven) eye movements, we decoded the bipolar voltage measures taken from the eye electrodes. The decoding approach was identical to that of the time course analysis of the EEG. The statistical analyses revealed no statistically significant clusters for which decoding accuracy was above zero.

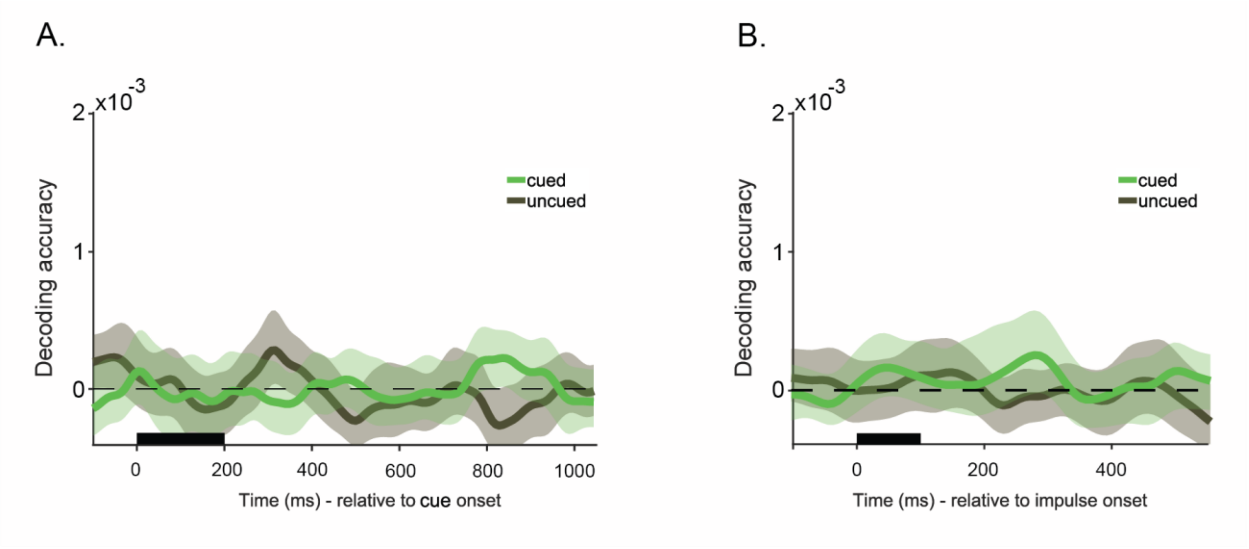

Figure S2. [Time-course decoding using eye electrodes], Related to Figure 3 and Figure 4. Mean decoding accuracy of the cued (green) and the uncued (black) items, relative to cue onset (A) and impulse onset (B) from the voltage measures from the bipolar eye electrodes. The black rectangular bar marks the presentation of the cue (A) and the impulse (B). Solid lines show the mean decoding accuracy over all trials and participants as a function of time. The shaded area around the mean marks the 95 % CI.

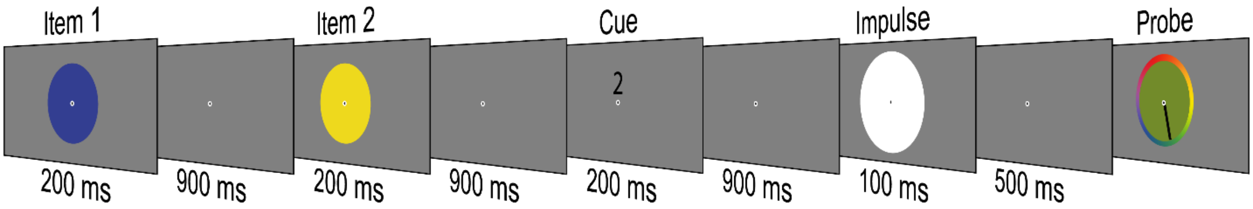

Figure S3. [Overview of a single trial in the experiment], Related to STAR Methods. In each trial, the colours of two sequentially presented disks would be memorized. A retro-cue then indicated the temporal position of the task-relevant memory item. Finally, the randomly-oriented probe was rotated by the participants with a joystick to report the task-relevant colour. The numbers below the stimulus displays reflect their duration in ms.

References

[1] van Ede, F., Chekroud, S. R., Stokes, M. G., & Nobre, A. C. (2019). Concurrent visual and motor selection during visual working memory guided action. *Nature Neuroscience*, *22*(3), 477-483. https://doi.org/10.1038/s41593-018-0335-6.

[2] Wolff, M. J., Kandemir, G., Stokes, M. G., & Akyürek, E. G. (2020). Unimodal and bimodal access to sensory working memories by auditory and visual impulses. Journal of Neuroscience, 40(3), 671–681. https://doi.org/10.1523/JNEUROSCI.1194-19.2019
